# Crucial Role of Selenium in the Virucidal Activity of Benzisoselenazol-3(2*H*)-ones and Related Diselenides

**DOI:** 10.3390/molecules15118214

**Published:** 2010-11-12

**Authors:** Magdalena Pietka-Ottlik, Piotr Potaczek, Egbert Piasecki, Jacek Mlochowski

**Affiliations:** 1 Faculty of Chemistry, Wroclaw University of Technology, Wybrzeze Wyspianskiego 27, 50-370 Wroclaw, Poland; E-Mails: piotr.potaczek@pwr.wroc.pl (P.P.); jacek.mlochowski@pwr.wroc.pl (J.M.); 2 Institute of Immunology and Experimental Therapy, Polish Academy of Sciences, R. Weigla 12, 53-114 Wroclaw, Poland.; E-Mail: piasecki@iitd.pan.wroc.pl (E.P.)

**Keywords:** organoselenium compounds, organosulfur compounds, isoindolin-1-ones, ebselen, antiviral activity

## Abstract

Various *N*-substituted benzisoselenazol-3(2*H*)-ones and their non-selenium-containing analogues have been synthesized and tested against selected viruses (HHV-1, EMCV and VSV) to determine the extent to which selenium plays a role in antiviral activity. The data presented here show that the presence of selenium is crucial for the antiviral properties of benzisoselenazol-3(2*H*)-ones since their isostructural analogues having different groups but lacking selenium either did not show any antiviral activity or their activity was substantially lower. The open-chain analogues of benzisoselenazol-3(2*H*)-ones—diselenides also exhibited high antiviral activity while selenides and disulfides were completely inactive towards model viruses.

Received: 29 October 2010; in revised form: 5 November 2010 / Accepted: 9 November 2010 / Published: 12 November 2010

## 1. Introduction

Over the last years, selenium-containing compounds have been proven to be promising antioxidants, enzyme mimics and inhibitors, immunomodulators, cytoprotectors, antitumor, anti-inflammatory, antihypertensive and antiinfectious agents [1]. Recently, considerable interest has been directed towards the antiviral properties of organoselenium compounds and in consequence some highly active benzisoselenazol-3(2*H*)-ones and diselenides have been successfully developed [2,3,4]. However, the mechanism of their antiviral action still remains unclear.

**Figure 1:**
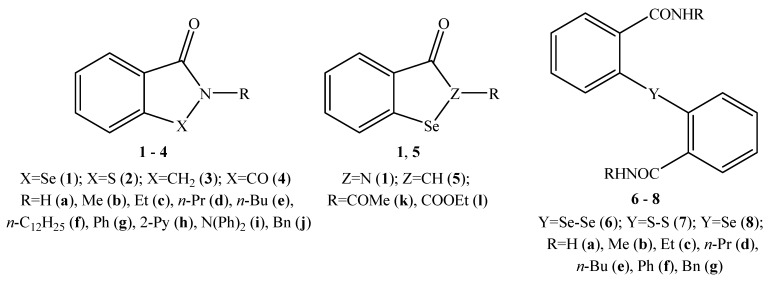
General formulas of tested compounds **1**–**8**.

In our study we wanted to determine whether selenium is crucial for antiviral properties of benzisoselenazol-3(2*H*)-ones **1**. For this purpose various *N*-substituted benzisoselenazol-3(2*H*)-ones **1** and their non-selenium-containing analogues **2**-**4** have been synthesized and tested *in vitro* against selected viruses (HHV-1 *human herpes virus type 1*, EMCV *Encephalomyocarditis virus* and VSV *Vesicular stomatitis virus*) (Figure 1). Since it is believed that the cleavage of the Se-N bond in benzisoselenazol-3(2*H*)-ones by thiols is responsible for their biological activity [1], we have compared the antiviral activity of benzisoselenazol-3(2*H*)-ones **1** and their analogues **5** that have the Se-C bond instead of the Se-N one to further understand the role of selenium in antivirals.

The second objective of our study was to compare the antiviral activity of benzisoselenazol-3(2*H*)-ones **1** and corresponding diselenides **6** and selenides **8**. Based on the circumstance that the antiviral properties of organoselenium compounds could be due to their ability to react with thiols we envisioned that diselenides which are open-chain analogues of benzisoselenazol-3(2*H*)-ones may also exhibit antiviral activity, while disulfides and selenides should be inactive. However, the antiviral activity of corresponding benzisoselenazol-3(2*H*)-ones and diselenides do not necessarily have to be the same value. Recently it has been shown that amide-based diselenides do not react with thiols as readily as e.g. amine-based diselenides do [5,6].

## 2. Results and Discussion

### 2.1. Synthesis

**Scheme 1:**
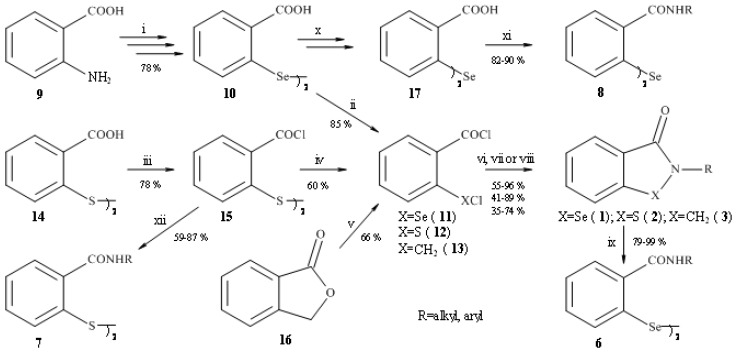
Synthesis of benzisoselenazol-3(2*H*)-ones (**1**) and related compounds.

The *N*-substituted benzisoselenazol-3(2*H*)-ones **1** have been prepared by the selenenylation-acylation of primary amines with 2-(chloroseleno)benzoyl chloride (**11**), obtained in a four-step synthesis starting from anthranilic acid (**9**), same way as reported in our previous works [3,4,7,8]. The same reaction of chloride **11** with corresponding *CH*-acids produced desired 3-hydroxybenzo[*b*]-selenophenes, which are more stable forms of benzo[*b*]selenophen-3(2*H*)-ones **5** (Scheme 1) [9,10]. 

For the synthesis of *N*-substituted benzisothiazol-3(2*H*)-ones **2** the analogous reaction of 2-(chlorosulfanyl)benzoyl chloride (**12**)**,** obtained in a two-step synthesis starting from 2,2’-dithiodibenzoic acid (**14**) [11], with corresponding primary amines has been adapted. This reaction has already been successfully used with some primary amines [12], hydrazine derivatives [13] and amino acids [14,15] as reactants. Our further studies have demonstrated that the reactions of chloride **12** with other *N*-nucleophiles, e.g., thiourea, thioacetamide, acetamide and p-methylbenzenesulfonamide also resulted in *N*-substituted benzisothiazol-3(2*H*)-ones, while reactions with *CH*-acids gave corresponding benzo[*b*]thiophen-3(2*H*)-ones or/and 3-hydroxybenzo[*b*]thiophenes [16]. 2,2’-Dithiodibenzoyl chloride (**15**) has been used to obtain various *N*-substituted disulfides **7** in the reaction with primary amines.

Since several *ortho*-disubstituted benzenes with both substituents being of electrophilic character, among them the abovementioned 2-(chloroseleno)benzoyl chloride (**11**) and 2-(chlorosulfanyl)benzoyl chloride (**12**), readily reacted with bisnucleophiles such as primary amines or activated methylene compounds to form a five-membered heterocyclic ring annulated onto the benzene moiety in a one-pot reaction, we decided to apply this approach to synthesize isoindolin-1-ones **3** (phthalimidines) as well. Although several synthetic approaches to particular isoindolin-1-ones have already been known, only a few of them such as reduction of one carbonyl group in phthalimides with zinc in acetic acid [17,18] or reaction of phthalide with primary amines, mostly under elevated pressure [19], are more general. The attempts to obtain *N*-substituted isoindolin-1-ones **3** by direct reaction of chloride **13** with primary amines were unsuccessful, because only *N*-acylation took place while the chloromethyl group remained unreactive [20,21]. The only exception was 2-(chloromethyl)-*N*-methylbenzamide, obtained from methylamine, that underwent cyclization to the corresponding *N*-methylisoindolin-1-one when treated with LDA at -78 °C [21]. In our study we have found that 2-(chlorophenyl)benzamide obtained from 2-(chloromethyl)benzoyl chloride (**13**) and aniline, treated with DBU easily cyclized to *N*-phenylisoindolin-l-one (**3g**) in almost quantitative yield. The same compound **3g** was obtained in one-pot procedure involving tandem acylation-alkylation of primary amines with 2-(chloromethyl)benzoyl chloride obtained from commercially available isobenzofuran-1(3*H*)-one treated with triphenylphosphine dichloride under microwave conditions (which allowed us to reduce the reaction time from 6 h to 5 min). Chloride **13** and aniline reacted in the presence of triethylamine for 2 h at room temperature and then DBU was added to the mixture and the reaction was continued for additional 2 h. This one-pot procedure was successfully extended on other *N*-substituted isoindolin-1-ones which were formed in good or moderate yields. Gaseous methylamine passed through the solution of chloride **13** in dichloromethane gave directly (without DBU) *N*-methylisoindolin-1-one (**3b**) while the reaction of **13** with gaseous ammonia led to the mixture of unstable compounds. Nevertheless unsubstituted isoindolin-1-one **3a** was obtained in moderate yield in a one-pot procedure involving alkoxylation of the chlorocarbonyl group of **13** to a carboxyester group, followed by acylation-alkylation of ammonia.

While the one-pot reaction of 2-(chloromethyl)benzoyl chloride (**13**) with amines in the presence of DBU was convienient for the synthesis of isoindolin-1-ones, the analogous reaction with *CH*-acids did not lead to expected cyclic 2,2-disubstituted 2,3-dihydroinden-1-ones and in all cases only acylation products were formed.

*N*-substituted phtalimides **4** have been prepared using standard procedures. Alkyl derivatives have been obtained in the reaction of potassium phtalimide with alkyl halides and phenyl phthalimide by heating phthalic anhydride and aniline.

The strategy for the synthesis of *N*-substituted bis(2-carbamoyl)phenyl diselenides **6** has been based on the reductive cleavage of the Se-N bond in corresponding benzisoselenazol-3(2*H*)-ones [7]. The selenides **8** have been prepared by the treatment of bis(2-chlorocarbonylphenyl) selenide, obtained from bis(2-carboxyphenyl) selenide **17**, with primary amines.

### 2.2. Virucidal Activity

The virucidal activity of compounds **1**-**8** has been determined *in vitro* towards HHV-1 (human herpes virus type 1, Herpesviridae, enveloped virus), EMCV (encephalomyocarditis virus, Picornaviridae, non-enveloped virus) and VSV (vesicular stomatitis virus, Rhabdoviridae, enveloped virus). The virus titer has been measured in human cell line A549 and the minimal inhibitory concentration MIC (μg/mL) has been determined. In the virucidal activity assay, compounds have been used in the non-toxic concentrations. The cytotoxicity of compounds has been assessed in the same tumor cell line (A549). The results are summarized in Table 1, Table 2 and Table 3.

From Table 1, it is evident that benzisoselenazol-3(2*H*)-ones **1** are much better antivirals than their non-selenium-containing analogues **2**–**4**. The majority of tested benzisoselenazol-3(2*H*)-ones **1** exhibited high antiviral activity towards HHV-1 (MIC in a range of 2.0-8.0 μg/mL) and EMCV (MIC in a range of 4.0-10.0 μg/mL) whereas their analogues either were completely inactive (MIC >1000 μg/mL for isoindolin-1-ones **3** and phtalimides **4**) or their activity was substantially lower (MIC in range of 80-1000 μg/mL for benzisothiazol-3(2*H*)-ones **2**) than the corresponding organo-selenium compounds. This clearly indicates that selenium plays crucial role in antiviral activity of benzisoselenazol-3(2*H*)-ones **1**. To verify whether this activity could be due to the presence of the labile Se-N bond, the antiviral activity of benzisoselenazol-3(2*H*)-ones and their analogues having the Se-C bond has been compared (Table 2). 3-Hydroxybenzo[*b*]selenophenes (more stable forms of benzo[*b*]selenophen-3(2*H*)-ones **5**) have been found to be inactive towards tested viruses (MIC >1000 μg/mL) while the corresponding benzisoselenazol-3(2*H*)-ones (**1k**, **1l**) exhibited high activity against HHV-1 and EMCV (MIC in a range of 4.0-10.0 μg/mL) ) and low activity against VSV (MIC = 600 μg/mL). Thus, it can be supposed that antiviral activity similarly to other biological activities of benzisoselenazol-3(2*H*)-ones is related to the presence of the Se-N bond which can undergo cleavage.

The majority of tested diselenides **6** exhibited virucidal activity towards HHV-1 and EMCV in the same range that corresponding benzisoselenazol-3(2*H*)-ones **1** (MIC in a range of 2.0-10.0 μg/mL). Only two diselenides **6f**, **6g** have been found inactive against EMCV. Like the benzisoselenazol-3(2*H*)-ones, none of tested diselenides exbibited antiviral activity against VSV. The replacement of the Se-Se bond in diselenides by the S-S one resulted in complete loss of antiviral activity. The corresponding selenides **8** have been found completely inactive against all tested viruses as well (Table 3). A comparison of antiviral activity of diselenides **6** and their abovementioned analogues **7**, **8** reveals that also in this case their activity can be due to the reaction with biologicaly important thiols. However, it is not confirmed in this case whether they are able to react directly with thiols or e.g. in the first step their cyclic analogues are formed to be active forms.

## 3. Experimental 

### 3.1. General

All reagents and solvents were purchased from Sigma-Aldrich or Fluka. Melting points were determined in open glass capillaries with an Electrothermal IA 9100 digital melting point apparatus. IR spectra were measured on a Perkin Elmer 2000 FT-IR spectrophotometer in KBr pellets or in thin films and peaks are reported in cm^-1^. Only representative absorptions are given. ^1^H-NMR, ^13^C-NMR and ^77^Se-NMR spectra were recorded in DMSO-d_6_ or CDCl_3_ on a 300 MHz Bruker DRX spectrometer (^1^H-NMR, ^13^C-NMR) or a 600 MHz Bruker AVII spectrometer (^77^Se-NMR). Chemical shifts are reported in ppm relative to TMS or dimethyl selenide. Reaction progress was monitored by a thin layer chromatography (TLC) on silica gel 60F254 coated aluminium TLC plates from Merck.

### 3.2. Benzisoselenazol-3(2H)-ones (**1**)

The synthesis and properties of compounds **1a**–**g** [4]**, 1l** [4], **1h** [3], **1j** [22] and **1k** [23] are given in references cited. Melting points and spectra were identical with the reported data.

*2-(N,N-Diphenylamino)benzisoselenazol-3(2H)-one* (**1i**). A solution of chloride **11** (1.27 g, 5.00 mmol) in dry dichloromethane (50 mL) was added dropwise over 30 min to a stirred solution of amine hydrochloride (5.00 mmol) and triethylamine (1.67g, 16.50 mmol) in dry dichloromethane (50 mL) in an ice/NaCl bath and the reaction was continued overnight, ending at room temperature. When the reaction was complete, the solvent was evaporated *in vacuo* and the crystalline residue was treated with water (100 mL) and stirred for 3 h. The insoluble solid was filtered off, washed with water and dried in the air. Crude product was purified by chromatography on silica gel with chloroform as the eluent and then recrystalized from ethyl acetate. 58% yield, yellow crystals, m.p. 154-156 °C, ^1^H-NMR (DMSO-d_6_) δ: 7.06-7.09 (m, 6H, ArH), 7.34 (t, 4H, *J* = 7.9 Hz, ArH), 7.46 (t, 1H, *J* = 7.4 Hz, ArH), 7.66-7.72 (m, 1H, ArH), 7.92 (d, 1H, *J* = 7.1 Hz, ArH), 8.04 (d, 1H, *J* = 8.0 Hz, ArH); ^77^Se-NMR (DMSO-d_6_) δ: 865; *ν*_max_ (KBr): 1633 (CO); Anal. Calcd for C_19_H_14_N_2_OSe: C, 62.47; H, 3.86; N, 7.67. Found: C, 62.46; H, 3.76; N, 7.62.

### 3.3. Benzisothiazol-3(2H)-ones **2**

2-(Chlorosulfanyl)benzoyl chloride (**12**) has been obtained according to the procedure described in references cited [11]. *Benzisothiazol-3(2H)-ones* **2a**–**c**. *Benzisothiazol-3(2H)-one* (**2a**), *2-methylbenzisothiazol-3(2H)-one* (**2b**), *2-ethylbenzisothiazol-3(2H)-one* (**2c**). General procedure: A vigrously stirred and cooled on ice/NaCl bath solution of 2-(chlorosulfanyl)benzoyl chloride (**12**, 0.83 g, 4.00 mmol) in dry dichloromethane (30 mL) was saturated by corresponding dry gaseous amine over 1 h. The reaction was continued for additional 1 h, finally in room temperature. After the reaction has finished, the mixture was washed with 5% HCl (3 × 50 mL) and then with water (2 × 50 mL). The organic layer was dried with anhydrous Na_2_SO_4_, the solvent was evaporated *in vacuo* and the residue was purified by silica gel chromatography (chloroform, and then ethyl acetate). Yields for **2a**–**c**: 85%, 76% and 81%, respectively. The compounds **2a** [24], **2b** [25], **2c** [26] are known compounds and their properties were identical with those given in references cited.

Benzisothiazol-3(2H)-ones **2d**–**j**. 2-n-Propylbenzisothiazol-3(2H)-one (**2d**), 2-n-butylbenzisothiazol-3(2H)-one (**2e**), 2-n-dodecylbenzisothiazol-3(2H)-one (**2f**), 2-phenylbenzisothiazol-3(2H)-one (**2g**), 2-(2-pyridyl)benzisothiazol-3(2H)-one (**2h**), 2-(N,N-diphenylamino)benzisothiazol-3(2H)-one (**2i**), 2-benzylbenzisothiazol-3(2H)-one (**2j**). General procedure: To a stirred on ice/NaCl bath solution of corresponding amine (16.50 mmol) for **2d**–**h**, **2j** or amine hydrochloride (5.00 mmol) and triethylamine (1.67 g, 16.50 mmol) for **2i** in dry dichloromethane (50 mL), a solution of chloride **12** (1.04 g, 5.00 mmol) in dry dichloromethane (50 mL) was dropped over 30 min. The reaction was continued for additional 4-16 h. After then the solvent was evaporated in vacuo and the crystalline residue was treated with water (100 mL) and stirred for 16 h. The insoluble solid was filtered off, washed with water and dried in the air. Crude products were recrystalized from hexane (**2f**), ethyl acetate (**2g**), methanol (**2h**, **2j**) or purified by chromatography on silica gel with chloroform as the eluent and then recrystalized from ethyl acetate (**2d**, **2e**, **2i**). Yields for **2d**–**j**: 77%, 81%, 81%, 73%, 88%, 41%, 89% respectively. The compounds **2d** [26], **2g** [24,26], **2h** [27] and **2j** [24,26] are known and their properties were in agreement with those given in references cited.

*2-n-Butylbenzisothiazol-3(2H)-one* (**2e**). 81% yield, yellow oil, ^1^H-NMR (CDCl_3_) δ: 0.96 (t, 3H, *J* = 7.6 Hz, CH_3_), 1.40 (m, 2H, (CH_2_)_2_CH_2_CH_3_), 1.74 (m, 2H, CH_2_CH_2_ CH_2_CH_3_), 3.89 (t, 2H, *J* = 7.6 Hz, CH_2_(CH_2_)_2_CH_3_), 7.38 (t, 1H, *J* = 7.8 Hz, ArH), 7.51-7.61 (m, 2H, ArH), 8.02 (d, 1H, *J* = 8.0 Hz, ArH); ^13^C-NMR (CDCl_3_) δ: 13.7, 19.8, 31.6, 43.7, 120.3, 124.9, 125.4, 126.6, 131.6, 140,2, 165.3; *ν*_max_ (film): 1660 (CO); Anal. Calcd for C_11_H_13_NOS: C, 63.74; H, 6.32; N, 6.76; S, 15.47. Found: C, 63.82; H, 6,36; N, 6.74; S, 15.52.

*2-n-Dodecylbenzisothiazol-3(2H)-one* (**2f**). 81% yield, white crystals, m.p. 39-40 °C, ^1^H-NMR (CDCl_3_) δ: 0.88 (t, 3H, *J* = 6.7 Hz, CH_3_), 1.26-1.35 (m, 18H, N(CH_2_)_2_(CH_2_)_9_CH_3_), 1.77 (m, 2H, NCH_2_CH_2_(CH_2_)_9_CH_3_), 3.89 (t, 2H, *J* = 7.2 Hz, NCH_2_(CH_2_)_10_CH_3_), 7.40 (t, 1H, *J* = 6.7 Hz, ArH), 7.51-7.62 (m, 2H, ArH), 8.04 (d, 1H, *J* = 7.8 Hz, ArH); ^13^C-NMR (CDCl_3_) δ: 14.1, 22.7, 26.6, 29.2, 29.2, 29.4, 29.5, 29.6, 31.9, 44.0, 120.3, 124.9, 125.4, 126.6, 131.6, 140.2, 165.3; *ν*_max_ (KBr): 1662 (CO); Anal. Calcd for C_19_H_29_NOS: C, 71.42; H, 9.15; N, 4.38; S, 10.04. Found: C, 71.48; H, 9.12; N, 4.44; S, 10.10%.

*2-(N,N-Diphenylamino)benzisothiazol-3(2H)-one* (**2i**). 41% yield, pale beige needles, m.p. 130 °C (decomp.), ^1^H-NMR (DMSO-d_6_) δ: 7.06-7.15 (m, 6H, ArH), 7.37 (t, 4H, *J* = 7.8 Hz, ArH), 7.48 (t, 1H, *J* = 7.5 Hz, ArH), 7.77 (t, 1H, *J* = 7.6 Hz, ArH), 7.95-8.00 (m, 2H, ArH); *ν*_max_ (KBr): 1680 (CO); Anal. Calcd for C_12_H_8_N_2_OS: C, 71.67; H, 4.43; N, 8.80; S, 10.07. Found: C, 71.61; H, 4.26; N, 8.73; S, 10.90.

### 3.4. Isoindolin-1-ones **3**

For the preparation of 2-(chloromethyl)benzoyl chloride (**13**) a vigorously stirred cooled on ice/NaCl bath solution of triphenylphosphine (26.23g, 0.10 mol) in dry dichloromethane (50 mL) was saturated by dry chlorine. Reaction progress was monitored by TLC. After the reaction has finished, isobenzofuran-1(3*H*)-one (13.41g, 0.10 mol) was added. The reaction was continued in microwave (5 min, 400 W). 2-(Chloromethyl)benzoyl chloride (**13**) was distilled off *in vacuo* from the reaction mixture (120 ºC/ 2 mmHg) as colourless oil. Yield 66%. Spectral data (^1^H-NMR, IR) were the same as these reported in the reference cited [28].

*Isoindolin-1-one* (**3a**). A solution of 2-(chloromethyl)benzoyl chloride (**13**, 0.94 g, 5.00 mmol) in ethanol (20 mL) was heated under reflux for 1 h. A solution of ammonia (20 mL) was added and the reaction was continued for an additional 1 h. After the reaction has finished the reaction mixture was extracted with dichloromethane (3 × 30 mL). The combined extracts were dried with anhydrous Na_2_SO_4_, the solvent was evaporated *in vacuo* and the residue was recrystalized from hexane-chloroform as white needles. Yield 35%.

*2-Methylisoindolin-1-one* (**3b**). A vigorously stirred solution of 2-(chloromethyl)benzoyl chloride (**13**, 0.94 g, 5.00 mmol) in dry dichloromethane (50 mL) cooled in an ice/NaCl bath was saturated by dry methylamine over 30 min. The reaction was continued for additional 24 h, finally in the room temperature. After the reaction has finished, dichloromethane was evaporated *in vacuo* and from the residue product was separated by silica gel chromatography (dichloromethane) and then recrystalized from hexane as white needles. Yield 74%.

Isoindolin-1-ones **3d**, **3g**–**j**. 2-n-Propylisoindolin-1-one (**3d**), 2-phenylisoindolin-1-one (**3g**), 2-(2-pyridyl)isoindolin-1-one (**3h**), 2-(N,N-diphenylamino)isoindolin-1-one (**3i**), 2-benzylisoindolin-1-one (**3j**). General procedure: To a stirred solution of amine (5.50 mmol) and triethylamine (0.56 g, 5.50 mmol) in dry dichloromethane (30 mL) the solution of chloride **13** (0.94 g, 5.00 mmol) in dry dichloromethane (20 mL) was added dropwise at room temperature for 30 min. After additional 2 h, DBU (1.54 g, 10.10 mmol) was added. The reaction was continued for additional 2 h at room temperature. After the reaction has finished, dichloromethane was evaporated in vacuo and the residue was separated by silica gel chromatography (dichloromethane) and then recrystalized from hexane-chloroform (3d, 3i, 3j), acetonitrile (3h) or hexane-ethyl acetate (3g). Yields for **3d**, **3g**–**j**: 61%, 69%, 60%, 65% and 59%, respectively.

The compounds **3a** [29], **3b** [30], **3d** [31], **3g** [32], **3h** [33], **3j** [32] are known and their properties were in agreement with those given in references cited.

*2-(N,N-diphenylamino)isoindolin-1-one* (**3i**). 65% yield, white needles, m.p. 168-170 °C, ^1^H-NMR (CDCl_3_) δ: 4.64 (s, 2H, CH_2_), 7.03-7.08 (m, 2H, ArH), 7.10-7.16 (m, 4H, ArH), 7.26-7.32 (m, 4H, ArH), 7.45 (d, 1H, *J* = 7.5 Hz, ArH), 7.51 (t, 1H, *J* = 7.3 Hz, ArH), 7.61 (dt, 1H, *J* = 6.3 and 1.2 Hz, ArH), 7.94 (d, 1H, *J* = 7.5 Hz, ArH); ^13^C-NMR (CDCl_3_) δ: 48.10, 119.81, 123.27, 123.40, 124.60, 128.38, 129.49, 131.07, 132.32, 139.72, 144.50, 166.86; *ν*_max_ (KBr): 1705 (CO); Anal. Calcd for C_20_H_16_N_2_O: C, 79.98; H, 5.37; N, 9.33. Found: C, 80.01; H, 5.39; N, 9.42.

### 3.5. Phthalimides **4**

Compound **4a** was commercially available (Sigma). It was used after recrystallization from ethanol. *Phthalimides* **4b**, **4d**. *N-Methylphthalimide* (**4b**), *N-n-propylphthalimide* (**4d**). General procedure: Potassium phthalimide (0.93 g; 5.00 mmol), alkyl iodide in excess and DMF (5 mL) were heated in reflux for 1h. Then the mixture was poured into water (30 mL) and extracted with dichloromethane (3 × 10 mL). After evaporating of the solvent, the crude product was recrystalized from methanol (**4b**) or ethanol (**4d**). Yields for **4b**, **4d**: 80%, 94%, respectively. Compounds **4b** [34] and **4d** [35] are known and their properties were in agreement with those given in references cited.

*N-Phenylphthalimide* (**4g**). *ortho*-Phthalic anhydride (0.74 g; 5.00 mmol) and aniline (0.47 g; 5.00 mmol) were heated in acetic acid (10 mL) for 1h. Then the mixture was cooled down and the insoluble product was filtered off, washed with water, dried and recrystallized from ethanol as colourless needles. Yield 74%. The chemical properties were in agreement with those given in references cited [36].

### 3.6. 3-Hydroxybenzo[b]selenophenes ***5***

The synthesis and properties of compounds **5k** [9] and **5l** [10] are given in references cited. Melting points and spectra were identical with the reported data.

### 3.7. Diselenides (**6**)

The synthesis of diselenides **6a**–**g** was carried out according to ref. [7]. The compounds **6a** [7], **6b** [7], **6f** [7] and **6g** [37] were previously reported and their properties were in agreement with those given in references cited.

*Bis[2-(N-ethylcarbamoyl)phenyl] diselenide* (**6c**). 79% yield, white powder, m.p. 210-212 °C, ^1^H-NMR (DMSO-d_6_) δ: 1.17 (t, 6H, *J* = 7.2 Hz, 2 × CH_3_), 3.30-3.38 (m, 4H, 2 × CH_3_), 7.29-7.40 (m, 4H, ArH), 7.69 (d, 2H, *J* = 7.5 Hz, ArH), 7.78 (dd, 2H, *J* = 7.4, 1.0 Hz, ArH), 8.71 (t, 2H, *J* = 4.9 Hz, NH); ^77^Se-NMR (DMSO-d_6_) δ: 455; Anal. Calcd for C_18_H_20_N_2_O_2_Se_2_: C, 47.59; H, 4.44; N, 6.17. Found: C, 47.86; H, 4.47; N, 6.14.

*Bis[2-(N-n-propylcarbamoyl)phenyl] diselenide* (**6d**). 79% yield, white powder, m.p. 223-224 °C, ^1^H-NMR (DMSO-d_6_) δ: 0.93 (t, 6H, *J* = 7.4 Hz, 2 × CH_3_), 1.52-1.64 (m, 4H, 2 × CH_2_CH_2_CH_3_), 3.23-3.30 (m, 4H, 2 × CH_2_CH_2_CH_3_), 7.29-7.40 (m, 4H, ArH), 7.69 (d, 2H, *J* = 7.8 Hz, ArH), 7.78 (dd, 2H, *J* = 7.4, 1.1 Hz, ArH), 8.71 (t, 2H, *J* = 5.3 Hz, NH); ^77^Se-NMR (DMSO-d_6_) δ: 455; Anal. Calcd for C_20_H_24_N_2_O_2_Se_2_: C, 49.80; H, 5.02; N, 5.81. Found: C, 49.93; H, 4.98; N, 5.83.

*Bis[2-(N-n-butylcarbamoyl)phenyl] diselenide* (**6e**). 89% yield, white powder, m.p. 181-183 °C, ^1^H-NMR (DMSO-d_6_) δ: 0.93 (t, 6H, *J* = 7.3 Hz, 2 × CH_3_), 1.31-1.43 (m, 4H, 2 × (CH_2_)_2_CH_2_CH_3_), 1.50-1.60 (m, 4H, 2 × CH_2_CH_2_CH_2_CH_3_), 3.26-3.33 (m, 4H, 2 × CH_2_(CH_2_)_2_CH_3_), 7.29-7.39 (m, 4H, ArH), 7.69 (d, 2H, *J* = 7.3 Hz, ArH), 7.77 (dd, 2H, *J* = 7.4, 1.1 Hz, ArH), 8.69 (t, 2H, *J* = 5.4 Hz, NH); ^77^Se-NMR (DMSO-d_6_) δ: 455; Anal. Calcd for C_22_H_28_N_2_O_2_Se_2_: C, 51.77; H, 5.53; N, 5.49. Found: C, 51.84; H, 5.52; N, 5.46. 

### 3.8. Disulfides **7**

The chloride **15** has been obtained according to the procedure described in references cited [11]. Disulfides **7a**–**g***. Bis(2-carbamoylphenyl disulfide* (**7a**), *bis[2-(N-methylcarbamoyl)phenyl] disulfide* (**7b**), *bis[2-(N-ethylcarbamoyl)phenyl] disulfide* (**7c**), *bis[2-(N-n-propylcarbamoyl)phenyl] disulfide* (**7d**), *bis[2-(N-n-butylcarbamoyl)phenyl] disulfide* (**7e**), *bis[2-(N-phenylcarbamoyl)phenyl] disulfide* (**7f**), *bis[2-(N-benzylcarbamoyl)phenyl] disulfide* (**7g**). General procedure: To a stirred solution of corresponding amine (8.80 mmol) in dry dichloromethane (25 mL), a solution of chloride **15** (0.69 g, 2.00 mmol) in dry dichloromethane (15 mL) was added dropwise over 30 min in room temperature. After the reaction has finished the solvent was evaporated *in vacuo* and the crystalline residue was treated with water (70 mL) and stirred for 4 h. The insoluble solid was filtered off, washed with water and dried in the air. Crude products were recrystalized from dioxane (**7a**, **7b**, **7f**), ethanol (**7c**–**e**) or ethyl acetate (**7g**). Yields for **7a**–**g**: 87%, 59%, 61%, 73%, 85%, 87%, 63% respectively. The compounds **7a** [24], **7b** [38], **7c**–**d** [39], **7e** [40] and **7f**–**g** [39] are known and their properties were in agreement with those given in references cited.

### 3.9. Selenides **8**

The chloride of **17** has been obtained according to the procedure described in references cited [41]. Selenides **8a**, **8b**, **8d**, **8f**. General procedure: To a stirred solution of corresponding amine (4.40 mmol) in dry acetonitrile (20 mL) (**8a**, **8b**) or dichloromethane (20 mL) (**8d**, **8f**), a solution of chloride of **17** (0.36 g, 1.00 mmol) in dry acetonitrile (10 mL) (**8a**, **8b**) or dichloromethane (10 mL) (**8d**, **8f**) was added dropwise over 30 min in room temperature. After the reaction has finished the solvent was evaporated *in vacuo* and the residue was treated with water (70 mL) and stirred for 4 h. The insoluble solid was filtered off, washed with water and dried in the air. Crude products were recrystalized from ethanol. 

*Bis(2-carbamoylphenyl) selenide* (**8a**). 89% yield, colourless crystals, m.p. 210-212 °C (ref. [41] 212-213 °C), ^1^H-NMR (DMSO-d_6_) δ: 7.22 (d, 2H, *J* = 7.2 Hz, ArH), 7.26-7.35 (m, 4H, ArH), 7.46 (bs, 2H, NH), 7.60 (d, 2H, *J* = 7.0 Hz, ArH), 7.94 (bs, 2H, NH); ^77^Se-NMR (DMSO-d_6_) δ: 448; *ν*_max_ (KBr): 3347, 3278, 3215 (NH), 1670, 1652 (CO); Anal. Calcd for C_14_H_12_N_2_O_2_Se: C, 52.68; H, 3.79; N, 8.78. Found: C, 51.50; H, 3.72; N, 8.52.

*Bis[2-(N-methylcarbamoyl)phenyl] selenide* (**8b**). 82% yield, colourless needles, m.p. 239.5-242 °C, ^1^H-NMR (DMSO-d_6_) δ: 2.84 (d, 6H, *J* = 4.6 Hz, 2 × CH_3_), 7.22-7.36 (m, 6H, ArH), 7.57 (d, 2H, *J* = 6.8 Hz, ArH), 7.93 (bs, 2H, NH); ^77^Se-NMR (DMSO-d_6_) δ: 440; *ν*_max_ (KBr): 3360, 3319 (NH), 1650, 1626 (CO); Anal. Calcd for C_16_H_16_N_2_O_2_Se: C, 55.34; H, 4.64; N, 8.07. Found: C, 54.13; H, 4.38; N, 7.84.

*Bis[2-(N-n-propylcarbamoyl)phenyl] selenide* (**8d**). 90% yield, white powder, m.p. 180-182 °C (decomp.), ^1^H-NMR (DMSO-d_6_) δ: 0.88 (t, 6H, *J* = 7.4 Hz, 2 × CH_3_), 1.43-1.55 (m, 4H, 2 × CH_2_CH_2_CH_3_), 3.13-3.19 (m, 4H, 2 × CH_2_CH_2_CH_3_), 7.21-7.36 (m, 6H, ArH), 7.53 (d, 2H, *J* = 7.1 Hz, ArH), 8.42 (t, 2H, *J* = 5.1 Hz, NH); ^77^Se-NMR (DMSO-d_6_) δ: 438; *ν*_max_ (KBr): 3350, 3261 (NH), 1653, 1627 (CO); Anal. Calcd for C_20_H_24_N_2_O_2_Se: C, 59.55; H, 6.00; N, 6.94. Found: C, 59.41; H, 6.01; N, 6.86.

*Bis[2-(N-phenylcarbamoyl)phenyl] selenide* (**8f**). 98% yield, white powder, m.p. 238-240 °C (decomp.), ^1^H-NMR (DMSO-d_6_) δ: 7.09 (t, 2H, *J* = 7.4 Hz, ArH), 7.31-7.35 (m, 6H, ArH), 7.38-7.40 (m, 2H, ArH), 7.42-7.45 (m, 2H, ArH), 7.69-7.72 (m, 6H, ArH), 10.43 (bs, 2H, NH); ^77^Se-NMR (DMSO-d_6_) δ: 434; *ν*_max_ (KBr): 3310 (NH), 1664 (CO); Anal. Calcd for C_26_H_20_N_2_O_2_Se: C, 66.24; H, 4.28; N, 5.94. Found: C, 65.70; H, 3.95; N, 5.85.

### 3.10. Cell cultures

Human lung adenocarcinoma A549 (ATCC 185) cells were maintained in Dulbecco’s modified Eagle’s medium supplemented with 2 mM L-glutammine, 5% heat-inactivated foetal bovine serum (FBS), 100 U/mL penicilin and 100 µg/mL streptomycin (all from Sigma) at 37 ºC in the atmosphere of 5% CO_2_ in air. The cells were plated at a density of 2 × 10^5^ cells/mL on 96-well plates (Costar-Corning) in 100 µL of fresh media the day before treatment with compounds.

### 3.11. Virucidal activity assay 

The compounds **1**-**8** at various concentrations (1-1,000 µg/mL) were incubated with following viruses: HHV-1 (human herpes virus type 1, Herpesviridae, enveloped virus), EMCV (encephalomyocarditis virus, Picornaviridae, non-enveloped virus) and VSV (vesicular stomatitis virus, Rhabdoviridae, enveloped virus). Viruses EMCV and VSV were used at the dose of 10^7^ TCID_50_/mL and HHV-1 at the dose of 10^5^ TCID_50_/mL. After 1 hour incubation at room temperature, the virus titer was measured in human cell line A549. Concentration of compound causing 1000 times decrease of virus titer was taken as minimal inhibitory concentration MIC (µg/mL). 

### 3.12. Cytotoxicity assay

The cytotoxicity of compounds was carried out with human lung adenocarcinoma cell line A549 (ATCC 185). The cells were incubated with serially diluted compounds for 48 hours at 37 ºC in the atmosphere of 5% CO_2_ in air. Then, the cultures were observed for cytotoxic effects. The minimal concentration of compound which was toxic to approximately 50% of cells was taken as TCCD_50_ (tissue culture cytotoxic dose). Final concentrations of compounds used in virucidal activity assay were below TCCD_50_ value that means they were not toxic for the cells.

## 4. Conclusions

Structural modifications that have been done in the isoselenazol-3(2*H*)-one ring of benzisoselenazol-3(2*H*)-ones **1** allowed us identify the fragment which is necessary for the antiviral activity of these compounds. The comparison of antiviral properties of benzisoselenazol-3(2*H*)-ones **1** and their isostructural analogues that do not contain selenium **2**–**4** clearly indicates the crucial role of this element for antiviral activity. The replacement of selenium by other atoms or functional groups drastically diminished the antiviral activity. While benzisoselenazol-3(2*H*)-ones **1** exhibited high antiviral activity directed towards HHV-1 and EMCV their non-selenium-containing analogues were completely inactive **3**, **4** or their activity was substantially lower (compounds **2**). The possible mechanism of action probably requires the cleavage of the Se-N bond since compounds having the Se-C bond instead of it did not exhibit any antiviral activity.

Our study has also demostrated that bis(2-carbamoylphenyl)diselenides **6** that are open-chain analogues of benzisoselenazol-3(2*H*)-ones **1** exhibit similar antiviral activity to the cyclic compounds, whereas the corresponding disulfides **7** and selenides **8** were completely inactive towards the model viruses. 

During our studies on the influence of selenium on antiviral activity a new synthetic approach to *N*-substituted isoindolin-1-ones **3** has been elaborated. It has been found that *N*-acylation of primary amines by chlorocarbonyl group of 2-(chloromethyl)benzoyl chloride (**13**), followed by their *N*-alkylation in the presence of DBU resulted in the pyrrolidone ring closure. Based on these reactions the simple, general one-pot procedure for synthesis of *N*-substituted isoindolin-1-ones **3** has been established.

## Figures and Tables

*Reagents and Conditions*: i. 1) NaNO _2_, HCl, H _2_O, 0-5 ºC; 2) Li _2_Se _2_, NaOH _aq_, -5-0 ºC; 3) HCl; ii. SOCl _2_ (excess), DMF (cat.), reflux; iii. SOCl _2_, reflux; iv. Cl_2_, CCl_4_, rt; v. Cl _2_P(Ph) _3_, MW (400W), 5 min; vi. R-NH _2_, CH_2_Cl_2_ or MeCN (X=Se); vii. R-NH _2_, CH_2_Cl_2_, -15 ºC (X=S); viii. 1) R-NH _2_, Et _3_N, CH_2_Cl_2_, rt, 2)DBU, rt (X=CH _2_); ix. N_2_H_4_, EtOH, reflux, x. 1) Zn, NaOH; 2) *o*-I-C _6_H _4_-COOH, K _2_CO _3_, Cu; xi. 1) SOCl _2_, benzene, reflux; 2) R-NH _2_, CH_2_Cl_2_ or MeCN; xii. R-NH _2_, CH_2_Cl_2_ or MeCN.

**Table 1:** Comparison of the virucidal activity of benzisoselenazol-3(2*H*)-ones **1** and their isostructural analogues **2**–**4**.

Compounds 1 - 4		MIC_HHV-1_				MIC_EMCV_				MIC_VSV_		
	^X^	Se	S	CH_2_	C=O	Se	S	CH_2_	C=O	Se, S	CH_2_	C=O
_R_												
H		8	>1000	>1000	>1000	4	>1000	>1000	>1000	>1000	>1000	>1000
Me		8	100	>1000	>1000	4	>1000	>1000	>1000	>1000	>1000	>1000
Et		8	400	ND	ND	4	>1000	ND	ND	>1000	ND	ND
*n*-Pr		6	100	>1000	>1000	6	>1000	>1000	>1000	>1000	>1000	>1000
*n*-Bu		6	80	ND	ND	6	>1000	ND	ND	>1000	ND	ND
*n*-C_12_H_25_		4	600	ND	ND	>1000	>1000	ND	ND	>1000	ND	ND
Ph		4	80	>1000	>1000	10	>1000	>1000	>1000	>1000	>1000	>1000
2-Py		4	>1000	>1000	ND	>1000	>1000	>1000	ND	>1000	>1000	ND
N(Ph)_2_		6	400	>1000	ND	>1000	>1000	>1000	ND	>1000	>1000	ND
Bn		2	60	>1000	ND	6	>1000	>1000	ND	>1000	>1000	ND
ACV		>1000^*^				ND				ND		

MIC – Minimal Inhibitory Concentration (µg/mL); ACV – acyclovir; ND – not determined; *Acyclovir was inactive in virucidal assay, but it inhibited viral replication at 20 µg/mL.

**Table 2:** Comparison of the virucidal activity of benzisoselenazol-3(2*H*)-ones **1** and their analogues having the Se-C bond instead of the Se-N bond **5**.

Compounds 1, 5		MIC_HHV-1_		MIC_EMCV_		MIC_VSV_	
	^Z^	N	CH	N	CH	N	CH
_R_							
COMe		4	>1000	6	>1000	600	>1000
COOEt		10	>1000	10	>1000	600	>1000

MIC = Minimal Inhibitory Concentration (μg/mL).

**Table 3:** Comparison of the virucidal activity of diselenides **6**, disulfides **7** and selenides **8**.

Compounds 6 - 8		MIC_HHV-1_			MIC_EMCV_			MIC_VSV_		
	^Y^	Se^_^Se	S^_^S	Se	Se^_^Se	S^_^S	Se	Se^_^Se	S^_^S	Se
_R_										
H		10	>1000	>1000	6	>1000	>1000	>1000	>1000	>1000
Me		2	>1000	>1000	6	>1000	>1000	>1000	>1000	>1000
Et		2	>1000	ND	4	>1000	ND	>1000	>1000	ND
*n*-Pr		6	>1000	>1000	40	>1000	>1000	>1000	>1000	>1000
*n*-Bu		10	>1000	ND	100	>1000	ND	>1000	>1000	ND
Ph		20	>1000	>1000	>1000	>1000	>1000	>1000	>1000	>1000
Bn		8	>1000	ND	>1000	>1000	ND	>1000	>1000	ND
ACV		>1000^*^			ND			ND		

MIC = Minimal Inhibitory Concentration (μg/mL); ACV – acyclovir; ND – not determined; *Acyclovir was inactive in virucidal assay, but it inhibited viral replication at 20 μg/mL.
